# Brief Report: Impact of COVID-19 on Individuals with ASD and Their Caregivers: A Perspective from the SPARK Cohort

**DOI:** 10.1007/s10803-020-04816-6

**Published:** 2021-01-02

**Authors:** L. Casey White, J. Kiely Law, Amy M. Daniels, Jaimie Toroney, Brianna Vernoia, Sabrina Xiao, The SPARK Consortium, Pamela Feliciano, Wendy K. Chung

**Affiliations:** 1 Simons Foundation, 160 5th Avenue, New York, NY 10010 USA; 2 Maryland Center for Developmental Disabilities, Kennedy Krieger Institute, Baltimore, MD 21211 USA; 3 Department of Pediatrics and Medicine, Columbia University Irving Medical Center, New York, NY 10032 USA

**Keywords:** Autism spectrum disorder, COVID-19, Services, Telehealth, Stress

## Abstract

The impact of the 2019 coronavirus pandemic (COVID-19) in the United States is unprecedented, with unknown implications for the autism community. We surveyed 3502 parents/caregivers of individuals with an autism spectrum disorder (ASD) enrolled in Simons Powering Autism Research for Knowledge (SPARK) and found that most individuals with ASD experienced significant, ongoing disruptions to therapies. While some services were adapted to telehealth format, most participants were not receiving such services at follow-up, and those who were reported minimal benefit. Children under age five had the most severely disrupted services and lowest reported benefit of telehealth adaptation. Caregivers also reported worsening ASD symptoms and moderate family distress. Strategies to support the ASD community should be immediately developed and implemented.

**Electronic supplementary material:**

The online version of this article (10.1007/s10803-020-04816-6) contains supplementary material, which is available to authorized users.

The 2019 coronavirus (COVID-19) pandemic has had an unprecedented impact on life around the world. In the United States, community transmission of COVID-19 was first detected in February 2020. By mid-March, there were reported cases in all 50 states (Centers for Disease Control 2020), and the World Health Organization declared that COVID-19 was a global pandemic (World Health Organization 2020). Many states issued stay-at-home directives and closed non-essential businesses by early April (e.g., CA: 3/19, NY: 3/22, WA: 3/23, D.C.: 4/1, FL: 4/3).

The closure of schools, clinics, and community programs puts children with autism spectrum disorder (ASD) at increased risk for negative outcomes. In addition to special education instruction, schools commonly provide critical services to children with ASD, including speech and language therapy (SLT), physical and occupational therapy (PT/OT), behavioral interventions and psychological supports (Koegel et al. 2012). Additional services targeting younger children, including interactive play-based therapies and Applied Behavioral Analysis (ABA), typically involve many hours (e.g. 20–40 h per week) of face-to-face intervention in the clinic or school and at home (Behavior Analyst Certification Board 2014). Children with ASD are also more likely than the general population to utilize clinic-based medical and mental health services (Cidav et al. 2012; Brookman-Frazee et al. 2011). Previous studies, before COVID-19, have established that the majority of school-age children with ASD (83–96%) in the United States were receiving some type of service or therapy (Monz et al. 2019; Zuckerman et al, 2017).

The delivery of ASD services and therapies in an online or remote format may partially ameliorate the impact of COVID-19. Prior research indicates that telehealth services may be an effective means of providing medical and psychiatric care to children with ASD (Bearss et al. 2017; Parsons et al. 2017) in addition to increased convenience for and empowerment of caregivers (Wallisch et al. 2019). Online parent-training programs may also be effective for therapies delivered in the home by a caregiver. A small study of parent-mediated therapies suggests that there are no differences in outcomes when parents are trained in-person versus via remote platforms (Hao et al. 2020) and that parent training delivered remotely can be effective at reducing problem behaviors in children with ASD (Lindgren et al. 2020). Overall, however, there is little evidence supporting the successful delivery of ASD services (including special education) in an online or remote setting.

The disruption of critical services and therapies, along with other changes in daily life, may result in worsening autism symptoms, increased behavioral challenges and decreased mental well-being for children with ASD. Prior research has shown that parents/caregivers of children with ASD have higher levels of stress than parents/caregivers of typically developing children (Hayes and Watson 2012) and with other neurodevelopmental disorders (Valicenti-McDermott et al. 2015). Furthermore, caregiver stress has been shown to increase with the severity of a child’s ASD symptoms (Argumedes et al. 2018; Shepherd et al. 2018). The COVID-19 pandemic may place parents/caregivers under additional stress and risk poorer mental health outcomes and family crisis.

Here we describe the impact of the COVID-19 pandemic on families of children with autism enrolled in the Simons Powering Autism Research for Knowledge (SPARK) research study. SPARK is a national, online research cohort of individuals with ASD and their family. We surveyed caregivers of children (and dependent adults) with ASD enrolled in SPARK to report disruptions in services and therapies for ASD, frequency of transition to online/telehealth and perceived benefit, and the overall emotional well-being of parents and children with ASD during the early months of the pandemic in the US.

## Methods

### Participants

Participants were recruited from SPARK, an online research cohort of individuals with ASD and their immediate family members (The SPARK Consortium 2018). The participants were caregivers (primarily parents) who initially registered their family into SPARK. All ASD diagnoses for dependent children in SPARK are parent/caregiver reported. Previous studies have demonstrated that parent/caregiver report diagnoses in an online research cohort are highly reliable (Daniels et al. 2012; Lee et al. 2010). For multiplex families, a single child was selected at random, and the parent was asked to complete the survey with this child in mind.

### Procedures

The SPARK study is approved by the Western Institutional Review Board (WIRB). All participants who consent to join the SPARK study agree to be contacted to complete additional questionnaires. Participants received an email invitation and up to two reminder emails from March 20, 2020 through April 1, 2020, for the first COVID-19 questionnaire (final participants completed on April 3, 2020), and April 23, 2020 through April 29, 2020 for the follow-up questionnaire (final participants completed on April 30, 2020). Only participants who completed the first questionnaire (N = 9030) were invited to complete the follow-up questionnaire. No incentive was provided for participation, but SPARK developed an infographic and article to share results with participants (Simons Foundation 2020).

### Measures

With the exception of the Brief Family Distress Scale (BFDS), all items in the questionnaires used by this study were written by the authors specifically to assess the impact of COVID-19 on the autism community. Questionnaires are included in supplemental files, with questions used as part of this study highlighted in bold text. The BFDS was used to measure the subjective experience of crisis. Developed for use in families of children with ASD, this single-item questionnaire asks parents to indicate their level of distress on a scale from 1 ‘everything is fine we are not in crisis’ to 10 ‘we are currently in crisis and it could not get any worse.’ Scores indicate caregiver impairment to effectively cope with or respond to current stressors. Impairment is categorized as ‘none’ (scores 1–3), ‘moderate’ (scores 4–5) or ‘marked’ (scores 6–10). Families with marked impairment are considered to be ‘near or in crisis.’ In a reference population of caregivers, the BFDS was normally distributed with a mean of 4.28 (SD = 1.65) (Weiss and Lunsky 2010).

### Data Analysis

Analyses included measures of central tendency (e.g. means and proportions) as well as tests for differences (e.g. chi-square and one-way analysis of variance tests) across the following age categories: 5 years and younger; 6 to 17 years; and 18 years and older. The Fisher’s exact test, as compared to the Chi-square test, was used whenever a cell size in any of the tabulations across age categories was less than five. The survey sample was also compared with the SPARK population on all sociodemographic and clinical characteristics to identify any significant differences. Data were analyzed using Stata version 12.1.

## Results

Among parents or caregivers who completed the first survey (N = 9030), 4369 completed the second survey. Among those, 760 reported receiving no services at baseline and were excluded. An additional 107 participants were excluded due to the inconsistency of their responses. In the follow-up survey, these participants reported receiving services at baseline. However, when they were asked whether services were impacted, their response was “Not applicable, my child doesn’t receive ASD services or therapies.” The final sample included 3,502 parents and caregivers of dependent children and adults with ASD who completed both baseline and follow-up surveys and reported receiving services at baseline, representing 39% of all families that completed the initial survey (Table 1).

**Table 1:** Participant characteristics (N = 3502)

Characteristic	
Parent/caregiver characteristics	
Age in years, mean (SD)	43.4 (8.8)
Sex at birth, N (%)	
Male	234 (7)
Female	3268 (93)
ASD diagnosis, N (%)	40 (1)
Child/dependent demographic and clinical characteristics	
Age in years, mean (SD)	11.8 (6.6)
Sex at birth, N (%)	
Male	2797 (80)
Female	705 (20)
Hispanic, N (%), n = 3249	510 (16)
Race, N (%), n = 3428	
White/Caucasian	2599 (80)
Black/African-American	145 (4)
Asian	74 (2)
Native-American	13 (0)
Native-Hawaiian/Pacific Islander	2 (0)
Other	98 (3)
Multiple	317 (10)
SCQ score, mean (SD), n = 2999	22.3 (7.0)
Ever intellectual disability, N (%)	739 (21)
Language level, N (%)	
No words/does not speak	465 (13)
Uses single words meaningfully	471 (13)
Combines three words together into short sentences (for example, to request)	591 (17)
Uses longer sentences of his/her own and is able to tell you something that happened	1975 (56)
Sociodemographic characteristics	
Household income, N (%), n = 3497	
$35 K and less	630 (19)
Between $36 and $65 K	682 (20)
Between $65 and $100 K	816 (23)
More than $100 K	1,101 (31)
Prefer not to answer	268 (8)
US Census region, N (%), n = 3379	
Northeast	662 (20)
Midwest	1044 (31)
South	814 (24)
West	859 (25)
Metropolitan classification, N (%), n = 3350	
Large central metropolitan area	927 (28)
Large fringe metropolitan area	993 (30)
Medium metropolitan area	691 (21)
Small metropolitan area	231 (10)
Micropolitan area	260 (8)
Non-core	158 (5)
Urban/rural status, N (%), n = 3350	
Urban	2932 (88)
Rural	418 (12)

Compared to invited participants, survey respondents were slightly older (43 years vs. 41 years, *p* < 0.001), more likely female (93% vs. 90%, *p* < 0.001) and White (80% vs. 68%, *p* < 0.001), and less likely Hispanic (16% vs. 20%, *p* < 0.001). Respondents were also more likely to come from the Northeastern (19% vs. 16%) than the Southern region United States (31% vs 37%, *p* < 0.001) and to report household incomes over $100 K (31% vs. 26%, *p* < 0.001) compared to non-respondents. There was no significant difference in child sex at birth comparing survey respondents to invited participants, however children included in this study were slightly older (11.8 vs. 11.4 years, *p* < 0.001). While mean Social Communication Questionnaire (SCQ) scores did not differ between the two groups, children included in the study were more likely to report intellectual disability (21% vs. 19%, p < 0.001) and use of longer sentences (56% vs. 53%, *p* = 0.003).

SPARK participants included in this study were comparable to those who completed only the first COVID survey, with few exceptions. Study participants were slightly older and children with ASD were slightly younger. Parents of ASD children were also more likely to report no speech or fewer words than those who participated in the first survey. Lastly, those included in this study had slightly higher overall household income and were more likely to come from a large metropolitan area. While the aforementioned differences were statistically significant (at p < 0.05), the magnitude of these differences was not considerable.

### Baseline Services and Disruptions Due to COVID-19

**Fig. 1:**
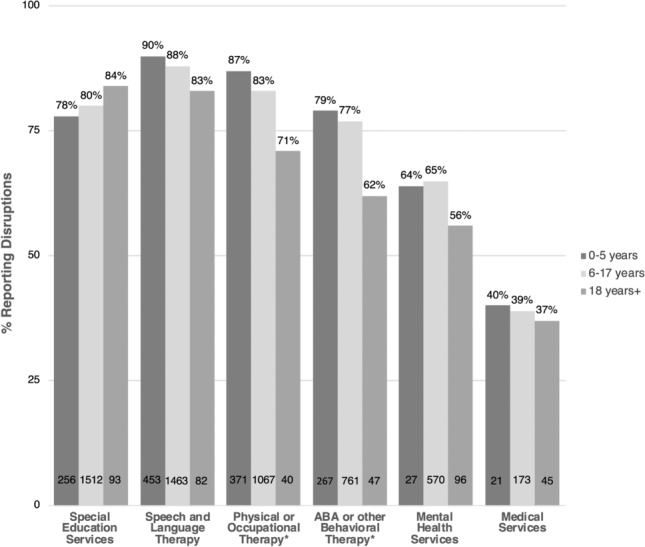
Children/Dependents with disrupted ASD services/therapies due to COVID-19. Percentages are among those receiving the service at baseline. The number of children/dependents with disrupted services for each age category is provided. Significant differences across age categories, as per Chi-square or Fisher’s exact test, are noted *p < .05

Irrespective of age category, the majority of parents and caregivers reported disruptions to special education (80%), speech and language therapy (SLT; 88%), physical and occupational therapies (PTOT; 84%), and applied behavior analysis (ABA; 77%) (see Fig. 1 for summary by age category). There were no significant differences across age groups in the most commonly received and disrupted services at baseline—special education, and SLT. In contrast, PTOT and ABA services were significantly more disrupted among the preschool-age group, as compared to school-age children and dependent adults (PTOT: 87% vs. 83% and 71%, respectively; ABA: 79% vs. 77% and 62%, respectively). Fewer than half of all parents/caregivers reported disruptions to medical services (39%).

### Use and Benefit of Remote and Online Services at Follow-Up

**Fig. 2:**
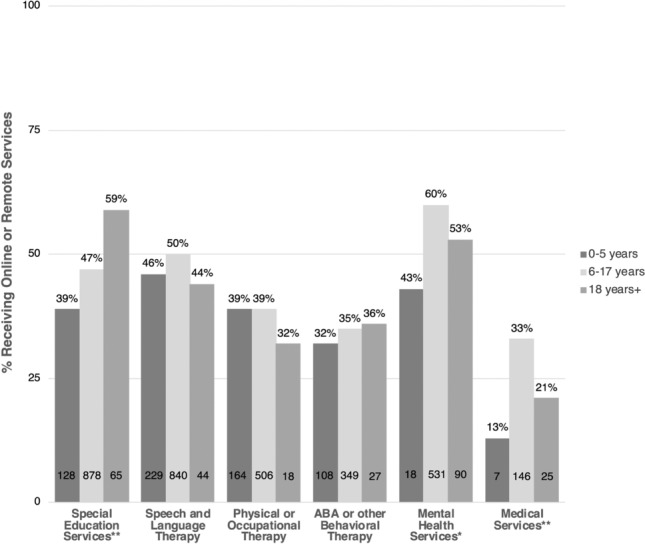
Children/dependents receiving online or remote ASD services/therapies at follow-up. Percentages are among those receiving the service at baseline. The number of children/dependents receiving online or remote services for each age category is provided. Significant differences across age categories, as per Chi-square or Fisher’s exact test, are noted. *p < .05, **p < .001

In general, the majority of individuals with ASD across all age groups did not receive online/adapted services in most categories at follow-up (Fig. 2).

**Fig. 3:**
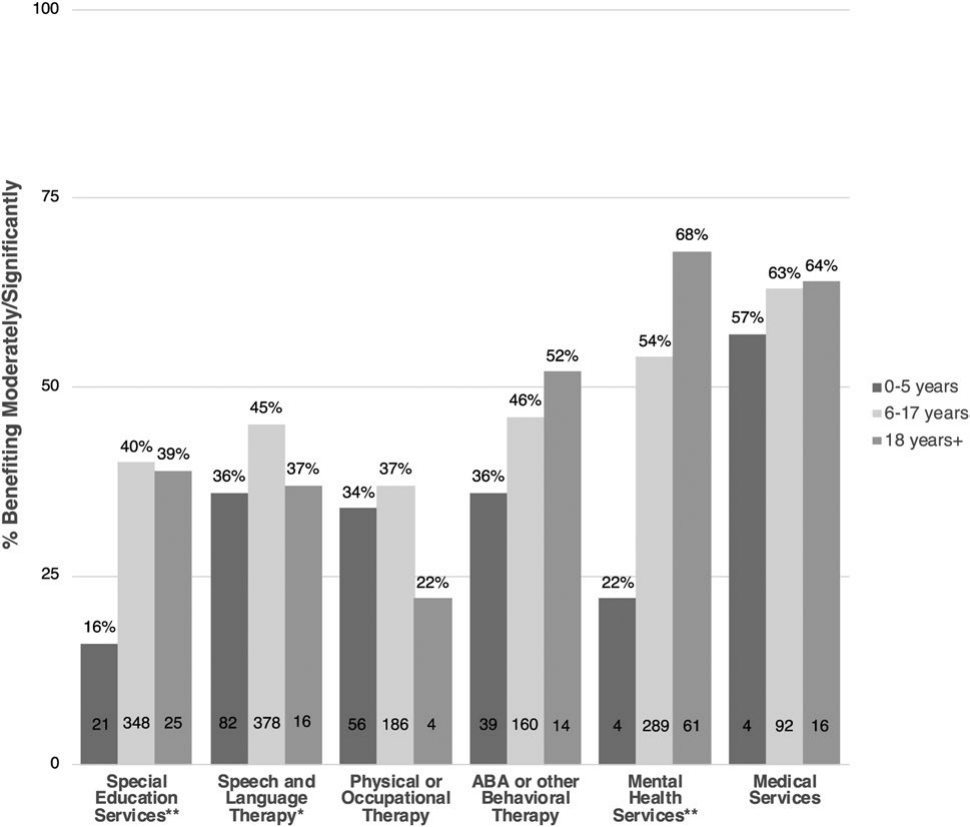
Children/dependents benefiting from online or remote services/therapies. Percentages are among those receiving online or remote services at follow-up. The number of children/dependents reporting moderate/significant benefits for each age category is provided. Significant differences across age categories, as per Chi-square or Fisher’s exact test, are noted. *p < .05, **p < .001

Among all remote and online services offered, only two—mental health and medical services, were reported by a majority of parents and caregivers of all children and dependents as significantly or moderately beneficial (55% and 63%, respectively). A considerably greater proportion of dependent adults (68%) reported significantly or moderately benefitting from online mental health services as compared to school-age children (54%) and preschool-age children (22%) (Fig. 3). School age children were significantly more likely to report a significant or moderate benefit to special education and SLT services offered remotely or online, as compared with preschool-age children and dependent adults (special education: 40% vs. 16% and 39%, respectively; SLT: 45% vs 36% and 37%, respectively).

Lastly, fewer than half of all respondents reported a significant or moderate benefit to remote or online PTOT (36%) and ABA (44%), and there were no significant differences across age groups.

### Impact on Families

Regarding impact of COVID-19 on child behaviors and parent stress and distress, just under two-thirds (64%) of all parents and caregivers reported that disruptions to services and therapies had severely or moderately impacted their children’s ASD symptoms, behaviors or challenges, and no differences were observed across age groups (see Table 2).

**Table 2:** Impact on families (N = 3502)

	All respondents (n = 3502)	5 years and younger (n = 603)	Between 6–17 years (n = 2488)	18 years and older (n = 411)	*P*-value
Child/dependent ASD symptoms (Q: To what extent have disruptions in services/therapies negatively impacted your child’s ASD symptoms, behaviors, or other challenges?)					
Severely/moderately	2151 (64)	382 (66)	1531 (64)	238 (62)	
Minimally/not at all	1203 (36)	201 (34)	856 (36)	146 (38)	.531
Parent stress (Q: To what extent do you feel stressed or overwhelmed by the disruptions in your child’s services/therapies?)					
Extremely/moderately	2475 (74)	470 (80)	1743 (73)	262 (68)	
Minimally/not at all	883 (26)	114 (20)	646 (27)	123 (32)	< .001
Brief Family Distress Scale					
Mean (SD)	3.6 (1.5)	3.6 (1.5)	3.7 (1.5)	3.4 (1.4)	< .001
No impairment	1747 (50)	314 (52)	1,199 (48)	234 (57)	
Moderate impairment	1442 (41)	239 (40)	1049 (42)	154 (38)	
Marked impairment	312 (9)	50 (8)	240 (10)	22 (5)	.003

While three-fourths of all parents reported extreme or moderate stress due to disruptions in their children’s services and therapies, it was greatest among preschool age children, (80%), followed by school age (73%) and dependent adult children (68%; *p* < 0.001). Parents of school age children were significantly more likely to endorse greater distress, measured both quantitatively and categorically, as compared to preschool-age and dependent adult children [mean 3.7 (SD 1.5) vs. 3.6 (1.5) and 3.4 (1.4), respectively]; categorically, as marked impairment: 10% vs. 8% and 5%, respectively).

## Discussion

The aim of this study was to examine the impact of COVID-19 on ASD services and caregiver perception of effectiveness of online adaptation, and impact of service disruption on families. Using previous studies as an approximation of service levels before COVID-19 (Monz et al. 2019; Zuckerman et al. 2017), this study found extensive disruptions to all types of ASD services due to COVID-19 at the beginning of the pandemic. Disruptions were more commonly reported for high intensity (e.g. daily or weekly) services such as ABA, special education, SLT, and PT/OT. Medical services were the least disrupted. This may be due to less frequent use, greater variability in the closing of medical offices (e.g. many remained open but with adapted procedures to limit person-to-person contact), and/or greater pre-COVID-19 familiarity with and/or more rapid transition to telehealth services. Most services were disrupted for a majority of individuals with ASD across all age groups, however, suggesting a widespread negative impact of COVID-19 on services and therapies. Individuals with autism and their families are an important vulnerable group for consideration of adapted services and additional support during similar emergency situations.

One solution to ameliorate social distancing impact on ASD services and therapies is to transition to telehealth or online platforms, which allow for continuity of care without increasing COVID-19 risk. At follow-up, all service types were adapted to telehealth delivery for some participants; however, for most services only a minority of dependent children/adults were receiving them in this manner. The exception was for mental health services, although this type of service is likely more easily adapted to telehealth delivery. Overall, the reported benefit of online and telehealth services was low (< 50%) across all service and therapy types and age groups except for medical and mental health services and dependent adults who receive ABA. Many traditional evidence-based therapies and services involve interactive play, peer-to-peer interactions, and reinforcers which are harder to deliver remotely. Families of preschool-aged children reported the least overall satisfaction with online and telehealth therapies, with a majority of participants reporting little to no perceived benefit for services such as ABA, SLT, and PT/OT. Adapting services to a telehealth format for this age group may be particularly challenging given the need to maintain the child’s attention on a computer screen. Many of these parents and children may also be relatively new to therapy, which would present distinct challenges when the therapist cannot be physically present with the family.

Disruption to services and therapies is an additional stressor on families with a child or dependent with ASD during an already stressful time due to the COVID-19 pandemic. A majority of dependents with ASD at all ages experienced worsening ASD symptoms. Increased ASD symptoms, some of which may be extreme (e.g. aggression, self-harm), along with skill loss or stagnation are likely to be stressful for both child and family. While a large majority of parents reported extreme to moderate stress specifically due to the loss of services and therapies, parents of preschool aged children were most stressed. This may be due to the emphasis on skill acquisition at this critical developmental period, with lost time being perceived as more impactful for this group. Parents/caregivers of school-aged children reported higher levels of overall distress than those of dependents of other ages. A higher percentage of older children have co-occurring mental health conditions, such as attention deficit/hyperactivity disorder (ADHD), anxiety, and disruptive behavior disorder (Neumeyer et al. 2019), that would be exacerbated by increased stress, which may contribute to challenges for this age group. It is notable that nearly 50% of families surveyed are endorsing distress at a level of moderate/marked impairment. This increased distress may put these families at higher risk of acute crisis and warrants monitoring by clinicians and other service providers in contact with these families as well as attempts to develop and share resources that may increase coping and reduce stress.

Overall, families with a child or dependent with ASD are experiencing major service disruptions and reporting feeling increased stress/distress due to the COVID-19 pandemic. Even when services and therapies are adapted into an online or telehealth format, many parents/caregivers report that these adaptations are not successful for a majority of therapy types. Additional research is needed to develop interventions that can be adapted and delivered remotely to various age groups. Such efforts may have the added benefit of helping to address disparities documented in rural and other underserved communities (Monz et al. 2019). Service providers should also consider solutions that allow for some in-person contact for critical therapies in a way that reduces risk, such as mask-wearing, contact tracing, and limiting in-person sessions to specific therapists/families, rather than rotating therapists or larger groups, to control the risk. Finally, clinicians and other service providers should monitor families with a dependent with ASD to watch for signs of worsening crisis risk and provide resources targeting areas of identified stress. Additional research and feedback from families may be needed to ensure that resources address the unique needs during a widespread emergency like the COVID-19 pandemic.

### Limitations

All responses are parent-reported and not assessed directly by a clinician or service provider. Because of the limited research in this area, the survey used in this study was not a validated measure, and therefore the results should be interpreted with caution. Studies used to approximate rates of service utilization among children with ASD before COVID did not involve the same participants, nor did they survey dependent adults with ASD, so they cannot be used as a true baseline comparison.

Additionally, our sample is slightly overrepresented for higher income families and majority white, with under representation of African American, Asian, Native American, and Native Hawaiian compared to data from the U.S. Census Bureau (Humes et al. 2011). While Hispanic respondents comprised 16% of the sample, which is representative of the US population, SPARK’s requirement of English language fluency means that non-English-speaking Latinx or other families would also not be represented. Compared to participants who only completed the first survey, parents who completed both surveys were slightly more likely to come from a large metropolitan area, had a slightly higher household income, and had a less verbal child. Evaluation of stress, distress, and areas of need would benefit from additional research with broader outreach to identify the groups most impacted and how to ameliorate negative effects and effectively intervene during this challenging time.

## Electronic supplementary material

Below is the link to the electronic supplementary material.

Electronic supplementary material 1 (PDF 466 kb)

Electronic supplementary material 2 (PDF 329 kb)

Electronic supplementary material 3 (PDF 328 kb)
