# Supplementary material for: Brief Report: Impact of COVID-19 on Individuals with ASD and Their Caregivers: A Perspective from the SPARK Cohort
Source: J Autism Dev Disord. 2021 Jan 2;51(10):3766–73. doi: 10.1007/s10803-020-04816-6 (PMC7775834; doi:10.1007/s10803-020-04816-6)
Supplement: Supplementary file 1 — Electronic supplementary material 1 (PDF 466 kb) [file 10803_2020_4816_MOESM1_ESM.pdf]

Impact of COVID-19 on ASD Services and Mental Health (Follow-up 1)

The following survey is a follow-up to the one you previously completed. It includes questions about the impact of the novel coronavirus (COVID-19) on your child's (or dependent's) ASD services and mental health. In particular, we are interested in finding out about what online and remote therapies are working, or not working, for your family. We hope these answers will help families improve their own experience and provide input we can share with advocacy groups and service providers. There are also questions about your mental health and about COVID-19 symptoms and testing.

Same as the survey you completed last time, you may skip any questions or stop answering these surveys at any time. Please note that no mental health professional will review any individual survey responses. If you are having emotional health difficulties, we strongly encourage you to (1) tell someone in your life about your feelings and concerns, and (2) to follow up with national mental health resources such as the National Suicide Prevention Lifeline at 1-800-273-8255.

For families who have more than one child with ASD registered in SPARK, we have randomly selected ONE child as the participant in this study. We are unable to change the selected child. **Please answer the survey questions with the selected child/dependent in mind.**

Section 1: Impact on ASD services and therapies

In the questions below, the phrase "ASD services or therapies" refers broadly to all services or therapies (e.g., medical, mental health, educational, and other supports) that your child/dependent might receive due to his/her ASD diagnosis.

Thinking back to January/February 2020 (before COVID-19 changes), was ChildFN receiving any ASD services or therapies?

- ◯ Yes
- ◯ No

Thinking back to January/February 2020 (before COVID-19 changes), which of the following *types* of <u>services or therapies</u> was ChildFN regularly receiving? *Check all that apply.*

- ☐ Early intervention services
- ☐ ABA services or other behavioral therapy
- ☐ Mental health services
- ☐ Medical services
- ☐ Speech and Language therapy
- ☐ Physical or Occupational therapy
- ☐ Special education services
- ☐ Other education services
- ☐ Recreational services
- ☐ Adult disability services
- ☐ Special transportation services
- ☐ Other

Thinking back to January/February 2020 (before COVID-19 changes), in which of the following <u>settings</u> did ChildFN receive ASD services or therapies? *Check all that apply.*

- ☐ School
- ☐ Professional clinic or office
- ☐ Daycare
- ☐ Residential programs
- ☐ Home (administered by visiting staff)
- ☐ Home (administered by parent or caregiver)
- ☐ Other

<u>Currently</u>, what percentage of ChildFN's services or therapies are disrupted due to COVID-19?

Click on the slider bar below to record your response. The color will change from gray to blue.

0 10 20 30 40 50 60 70 80 90 100

<u>Currently</u>, what percentage of ChildFN's services or therapies have been successfully adapted or modified?

Click on the slider bar below to record your response. The color will change from gray to blue.

0 10 20 30 40 50 60 70 80 90 100

Currently, what percentage of ChildFN's education, services, or therapies are you now responsible for delivering (do not consider education, services, or therapies that you were responsible for delivering prior to COVID-19 changes)?

Click on the slider bar below to record your response. The color will change from gray to blue.

0 10 20 30 40 50 60 70 80 90 100

How well are you coping with the added responsibility of delivering ChildFN's education, services, or therapies?

- Completely
- Moderately
- Minimally
- Not at all

Currently, many school systems and professionals are implementing online or remote delivery of services (tele-health) and therapies. Is ChildFN currently receiving any services or therapies using this approach?

- Yes
- No

Which services or therapies have changed to online or remote delivery?

- Early intervention services
- ABA services or other behavioral therapy
- Mental health services
- Medical services
- Speech and Language therapy
- Physical or Occupational therapy
- Special education services
- Other education services
- Recreational services
- Adult disability services
- Other

Please specify "other" services:

To what extent do you think ChildFN is benefitting from early intervention services delivered online or remotely?

- Significantly
- Moderately
- Minimally
- Not at all

To what extent do you think ChildFN is benefitting from ABA services or other behavioral therapy delivered online or remotely?

- Significantly
- Moderately
- Minimally
- Not at all

To what extent do you think ChildFN is benefitting from mental health services delivered online or remotely?

- Significantly
- Moderately
- Minimally
- Not at all

To what extent do you think ChildFN is benefitting from medical services delivered online or remotely?

- Significantly
- Moderately
- Minimally
- Not at all

To what extent do you think ChildFN is benefitting from speech and language therapy delivered online or remotely?

- Significantly
- Moderately
- Minimally
- Not at all

To what extent do you think ChildFN is benefitting from physical or occupational therapy delivered online or remotely?

- ○ Significantly
- ○ Moderately
- ○ Minimally
- ○ Not at all

To what extent do you think ChildFN is benefitting from special education services delivered online or remotely?

- ○ Significantly
- ○ Moderately
- ○ Minimally
- ○ Not at all

To what extent do you think ChildFN is benefitting from other education services delivered online or remotely?

- ○ Significantly
- ○ Moderately
- ○ Minimally
- ○ Not at all

To what extent do you think ChildFN is benefitting from recreational services delivered online or remotely?

- ○ Significantly
- ○ Moderately
- ○ Minimally
- ○ Not at all

To what extent do you think ChildFN is benefitting from adult disablity services delivered online or remotely?

- ○ Significantly
- ○ Moderately
- ○ Minimally
- ○ Not at all

To what extent do you think ChildFN is benefitting from other services delivered online or remotely?

- ○ Significantly
- ○ Moderately
- ○ Minimally
- ○ Not at all

When services or therapies are delivered online or remotely, what is or is not working?

NEXT

You may close this window at any time to **Save and Exit**.

Any data entered on this page will be lost. All previous pages will be saved.

SPARK

Simons Powering Autism Research

Impact of COVID-19 on ASD Services and Mental Health (Follow-up 1)

Section 2: Impact on parent employment and financial stability

Which of the following options best describes your employment status in January/February 2020 (before COVID-19 changes)?

- ○ Full-time
- ○ Part-time
- ○ Self-Employed
- ○ Other
- ○ Did not have paid work

To what extent has your employment status been negatively impacted due to COVID-19?

- ○ Significantly
- ○ Moderately
- ○ Minimally
- ○ Not at all

What sorts of changes are you experiencing with regard to employment due to COVID-19? *Check all that apply.*

- ☐ Working remotely all or part of the time
- ☐ Working, but with reduced hours
- ☐ Furloughed with pay (full or partial)
- ☐ Furloughed without pay
- ☐ Laid off
- ☐ High risk of being exposed to the novel coronavirus while working

- [ ] Working at home while taking care of dependent children
- [ ] Other

How well are you coping with changes in your employment?

- ( ) Completely
- ( ) Moderately
- ( ) Minimally
- ( ) Not at all

How would you describe the <u>financial stability</u> of your household in January/February 2020 (before COVID-19 changes)?

- ( ) Excellent
- ( ) Very good
- ( ) Good
- ( ) Fair
- ( ) Poor

To what extent has your household's financial stability been negatively impacted by COVID-19?

- ( ) Significantly
- ( ) Moderately
- ( ) Minimally
- ( ) Not at all

In 2019, what was your total annual household income?

- ( ) Less than $20,000
- ( ) $21,000 - $35,000
- ( ) $36,000 - $50,000
- ( ) $51,000 - $65,000
- ( ) $66,000 - $80,000
- ( ) $81,000 - $100,000
- ( ) $101,000 - $130,000
- ( ) $131,000 - $160,000
- ( ) Over $161,000
- ( ) Prefer not to answer

PREVIOUS NEXT

You may close this window at any time to **Save and Exit.**

Any data entered on this page will be lost. All previous pages will be saved.

Impact of COVID-19 on ASD Services and Mental Health (Follow-up 1)

Section 3: Impact on child, parent and family well-being

During the past week, how would you describe ChildFN's emotional or mental health?

- ◯ Excellent
- ◯ Very good
- ◯ Good
- ◯ Fair
- ◯ Poor

During the past week, how would you describe your own emotional or mental health?

- ◯ Excellent
- ◯ Very good
- ◯ Good
- ◯ Fair
- ◯ Poor

Which of the following activities have you been doing to cope with COVID-19 related life changes in the last month? *Check all that apply.*

- ☐ Exercise or getting outdoors to enjoy nature
- ☐ Practicing meditation or mindfulness
- ☐ Listening to music
- ☐ Drinking alcohol
- ☐ Taking a recreational drug, or misusing prescription medicine
- ☐ Smoking, vaping or chewing tobacco

- [ ] Seeking counseling
- [ ] Seeking support from social network
- [ ] Pursuing hobbies or other creative activities
- [ ] Gardening or making home repairs
- [ ] Watching TV, movies, or other media
- [ ] Reading
- [ ] Spiritual activities or support
- [ ] Learning a new skill
- [ ] Eating more or less than normal
- [ ] Sleeping more or less than normal
- [ ] Spending time with members of my household
- [ ] Spending time with a pet
- Other
- [ ] None

Please specify "other" activities:

<u>In the past week</u>, how often have you felt nervous, anxious, or on edge?

- ○ Rarely or none of the time (less than 1 day)
- ○ Some or a little of the time (1-2 days)
- ○ Occasionally or a moderate amount of time (3-4 days)
- ○ Most or all of the time (5-7 days)

<u>In the past week</u>, how often have you felt depressed?

- ○ Rarely or none of the time (less than 1 day)
- ○ Some or a little of the time (1-2 days)
- ○ Occasionally or a moderate amount of time (3-4 days)
- ○ Most or all of the time (5-7 days)

<u>In the past week</u>, how often have you felt lonely?

- ○ Rarely or none of the time (less than 1 day)
- ○ Some or a little of the time (1-2 days)
- ○ Occasionally or a moderate amount of time (3-4 days)
- ○ Most or all of the time (5-7 days)

<u>In the past week</u>, how often have you felt hopeful about the future?

- Rarely or none of the time (less than 1 day)
- Some or a little of the time (1-2 days)
- Occasionally or a moderate amount of time (3-4 days)
- Most or all of the time (5-7 days)

In the past week, how often have you had physical reactions, such as sweating, trouble breathing, nausea, or a pounding heart, when thinking about your experience (e.g., social distancing, loss of income/work, concerns about infection) with the coronavirus/COVID-19 pandemic?

- Rarely or none of the time (less than 1 day)
- Some or a little of the time (1-2 days)
- Occasionally or a moderate amount of time (3-4 days)
- Most or all of the time (5-7 days)

Overall, how concerned are you about the impact of COVID-19 on your family or household?

- Extremely
- Moderately
- Minimally
- Not at all

Please indicate where you and your family currently are right now, in terms of crisis, by selecting one of the following statements:

- 1 = Everything is fine, my family and I are not in crisis at all
- 2 = Everything is fine, but sometimes we have our difficulties
- 3 = Things are sometimes stressful, but we can deal with problems if they arise
- 4 = Things are often stressful, but we are managing to deal with problems when they arise
- 5 = Things are very stressful, but we are getting by with a lot of effort
- 6 = We have to work extremely hard every moment of every day to avoid having a crisis
- 7 = We won't be able to handle things soon. If one more thing goes wrong, we will be in crisis
- 8 = We are currently in crisis, but are dealing with it ourselves
- 9 = We are currently in crisis, and have asked for help from crisis services (Emergency room, hospital, community crisis support)
- 10 = We are currently in crisis, and it could not get any worse

PREVIOUS NEXT

You may close this window at any time to **Save and Exit**.

Any data entered on this page will be lost. All previous pages will be saved.

SPARK

Simons Powering Autism Research

Impact of COVID-19 on ASD Services and Mental Health (Follow-up 1)

Section 4: Other information

Which of these recommendations did you follow during the past week? *Check all that apply.*

- [ ] Avoid crowded places
- [ ] Avoid public places
- [ ] Keep your distance from others (6 feet)
- [ ] Change school or work arrangements
- [ ] Quarantine yourself if you have symptoms
- [ ] None

In the past week, has anyone in your household had symptoms that were concerning for COVID-19?

- ○ Yes
- ○ No

Has anyone in your family or household tested positive for COVID-19?

- ○ Yes
- ○ No

Do you know anyone personally (outside of your family or household) who has tested positive for COVID-19?

- ○ Yes
- ○ No

Do you know anyone personally who has been hospitalized from COVID-19?

- ◯ Yes
- ◯ No

Do you know anyone personally who has died from COVID-19?

- ◯ Yes
- ◯ No

PREVIOUS NEXT

You may close this window at any time to **Save and Exit**.

Any data entered on this page will be lost. All previous pages will be saved.

Impact of COVID-19 on ASD Services and Mental Health (Follow-up 1)

Section 5: Additional Comments

During the past month, what have been the biggest challenges for ChildFN and for your family?

During the past month, what have been the biggest successes for ChildFN and for your family?

PREVIOUS SUBMIT

You may close this window at any time to **Save and Exit**.

Any data entered on this page will be lost. All previous pages will be saved.
