# Supplementary material for: Brief Report: Impact of COVID-19 on Individuals with ASD and Their Caregivers: A Perspective from the SPARK Cohort
Source: J Autism Dev Disord. 2021 Jan 2;51(10):3766–73. doi: 10.1007/s10803-020-04816-6 (PMC7775834; doi:10.1007/s10803-020-04816-6)
Supplement: Supplementary file 3 — Electronic supplementary material 3 (PDF 328 kb) [file 10803_2020_4816_MOESM3_ESM.pdf]

# Impact of COVID-19 on ASD Services and Mental Health

The following survey includes questions about the impact of the novel coronavirus (COVID-19) on your child's (or dependent's) ASD services and mental health. There are also questions about your mental health and your family's experiences as related to COVID-19 policies and news.

For families who have more than one child with ASD registered in SPARK, we have randomly selected ONE child as the participant in this study. We are unable to change the selected child. **Please answer the survey questions with the selected child/dependent in mind.**

**Section 1: About your child/dependent's ASD services**

In the questions below, the phrase "ASD services or therapies" refers broadly to all services or therapies (e.g., medical, mental health, educational, and other supports) that your child/dependent might receive due to his/her ASD diagnosis.

**In the past week, to what extent have ChildFN's ASD services or therapies been disrupted due to COVID-19?**

- ◯ Severely
- ◯ Moderately
- ◯ Minimally
- ◯ Not at all
- ◯ Not applicable; My child doesn't receive ASD services or therapies

**Which of the following *settings* for services or therapies have been disrupted? *Select all that apply.***

- ☐ School

- [ ] Professional clinic or office
- [ ] Daycare
- [ ] Residential programs
- [ ] Home (administered by visiting staff)
- [ ] Home (administered by parent or caregiver)

Other

**Please specify "other" settings:**

**Which of the following best describes the current status of ChildFN's school (includes all school levels)? *If not in school, select "not applicable".***

- ◯ Closed due to COVID-19
- ◯ Closed due to previously scheduled spring break or other reason
- ◯ Open
- ◯ Not applicable; not in school

**Which of the following *types* of services or therapies have been disrupted? *Select all that apply.***

- [ ] Early intervention services
- [ ] ABA services or other behavioral therapy
- [ ] Mental health services
- [ ] Medical services
- [ ] Speech and Language therapy
- [ ] Physical or Occupational therapy
- [ ] Special education services
- [ ] Other education services
- [ ] Recreational services
- [ ] Adult disability services
- [ ] Special transportation services

Other

**Please specify "other" types of services or therapies:**

**Overall, what percentage of ChildFN's services or therapies have been disrupted due to COVID-19?**

0 10 20 30 40 50 60 70 80 90 100

**Overall, what percentage of ChildFN's services or therapies have been successfully adapted or modified in response to the current disruptions?**

0 10 20 30 40 50 60 70 80 90 100

**To what extent have disruptions in services or therapies negatively impacted ChildFN's autism symptoms, behaviors or other challenges?**

- ◯ Severely
- ◯ Moderately
- ◯ Minimally
- ◯ Not at all

**To what extent do you feel stressed or overwhelmed by the disruption in ChildFN's services or therapies?**

- ◯ Extremely
- ◯ Moderately
- ◯ Minimally
- ◯ Not at all

**Currently, many school systems and professionals are implementing online or remote delivery of services and therapies. Is ChildFN currently receiving any services or therapies using this approach?**

- ◯ Yes
- ◯ No

**To what extent do you think ChildFN is benefitting from services or therapies delivered online or remotely?**

- ◯ Significantly
- ◯ Moderately
- ◯ Minimally
- ◯ Not at all

**We are interested in hearing your ideas. Do you have any suggestions for ways that medical or service professionals could help meet your child/dependent's service or therapy needs during this time?**

NEXT

You may close this window at any time to **Save and Exit**.

*Any data entered on this page will be lost. All previous pages will be saved.*

SPARK

Simons Powering Autism Research

# Impact of COVID-19 on ASD Services and Mental Health

## Section 2: About your child/dependent's emotional or mental health

**To what extent do you think ChildFN understands information or news related to COVID-19?**

- ◯ Completely
- ◯ Moderately
- ◯ Minimally
- ◯ Not at all

**During the past week, how would you describe ChildFN's emotional or mental health?**

- ◯ Excellent
- ◯ Very good
- ◯ Good
- ◯ Fair
- ◯ Poor

**To what extent has ChildFN's emotional or mental health been negatively impacted due to COVID-19 concerns?**

- ◯ Severely
- ◯ Moderately
- ◯ Minimally
- ◯ Not at all
- ◯ Don't know

PREVIOUS | NEXT

You may close this window at any time to **Save and Exit.**

*Any data entered on this page will be lost. All previous pages will be saved.*

*Any data entered on this page will be lost. All previous pages will be saved.*

SPARK

Simons Powering Autism Research

# Impact of COVID-19 on ASD Services and Mental Health

## Section 3: About your emotional or mental health

The following set of questions are asking about YOU and how well YOU are handling the current circumstances.

**During the past week, how would you describe your own emotional or mental health?**

- ◯ Excellent
- ◯ Very good
- ◯ Good
- ◯ Fair
- ◯ Poor

**To what extent has your own emotional or mental health been negatively impacted by COVID-19?**

- ◯ Severely
- ◯ Moderately
- ◯ Minimally
- ◯ Not at all

**<u>In the past week</u>, how often have you felt nervous, anxious, or on edge?**

- ◯ Rarely or none of the time (less than 1 day)
- ◯ Some or a little of the time (1-2 days)
- ◯ Occasionally or a moderate amount of time (3-4 days)
- ◯ Most or all of the time (5-7 days)

**<u>In the past week</u>, how often have you felt depressed?**

- Rarely or none of the time (less than 1 day)
- Some or a little of the time (1-2 days)
- Occasionally or a moderate amount of time (3-4 days)
- Most or all of the time (5-7 days)

**In the past week, how often have you felt lonely?**

- Rarely or none of the time (less than 1 day)
- Some or a little of the time (1-2 days)
- Occasionally or a moderate amount of time (3-4 days)
- Most or all of the time (5-7 days)

**In the past week, how often have you felt hopeful about the future?**

- Rarely or none of the time (less than 1 day)
- Some or a little of the time (1-2 days)
- Occasionally or a moderate amount of time (3-4 days)
- Most or all of the time (5-7 days)

**In the past week, how often have you had physical reactions, such as sweating, trouble breathing, nausea, or a pounding heart, when thinking about your experience (e.g., social distancing, loss of income/work, concerns about infection) with the coronavirus/COVID-19 pandemic?**

- Rarely or none of the time (less than 1 day)
- Some or a little of the time (1-2 days)
- Occasionally or a moderate amount of time (3-4 days)
- Most or all of the time (5-7 days)

**Has a doctor or other healthcare provider EVER told you that you have a mental health condition?**

- Yes
- No

PREVIOUS NEXT

You may close this window at any time to **Save and Exit.**

*Any data entered on this page will be lost. All previous pages will be saved.*

# Impact of COVID-19 on ASD Services and Mental Health

## Section 4: Other information

**What is your zip code?**

**Which of these recommendations did you follow during the past week?** ***Select all that apply.***

- [ ] Avoid crowded places
- [ ] Avoid public places
- [ ] Keep your distance from others (6 feet)
- [ ] Change school or work arrangements
- [ ] Quarantine yourself if you have symptoms
- [ ] None

**In the past week, has anyone in your household had symptoms that were concerning for COVID-19?**

- ( ) Yes
- ( ) No

**Has anyone in your family or household tested positive for COVID-19?**

- ( ) Yes
- ( ) No

**Do you know anyone personally (outside of your family or household) who has tested positive for COVID-19?**

- ( ) Yes
- ( ) No

**Do you know anyone personally who has been hospitalized from COVID-19?**

- ◯ Yes
- ◯ No

**Do you know anyone personally who has died from COVID-19?**

- ◯ Yes
- ◯ No

**Overall, how concerned are you about the impact of COVID-19 on your family or household?**

- ◯ Extremely
- ◯ Moderately
- ◯ Minimally
- ◯ Not at all

**Is there any additional information you would like to share about the impact of COVID-19 on you, your child or your family?** ***For instance, what are you currently doing, or thinking about doing, to cope with or adapt to the changes imposed on you and your family due to COVID-19?***

**Do you have any successful strategies that you would like to share? What's working for you and your family?**

PREVIOUS SUBMIT

You may close this window at any time to **Save and Exit**.

*Any data entered on this page will be lost. All previous pages will be saved.*
